# Terahertz and mid-infrared reflectance of epitaxial graphene

**DOI:** 10.1038/srep24301

**Published:** 2016-04-22

**Authors:** Cristiane N. Santos, Frédéric Joucken, Domingos De Sousa Meneses, Patrick Echegut, Jessica Campos-Delgado, Pierre Louette, Jean-Pierre Raskin, Benoit Hackens

**Affiliations:** 1 IMCN/NAPS Université catholique de Louvain, Louvain-la-Neuve 1348, Belgium; 2 Research Center in Physics of Matter and Radiation (PMR), University of Namur (UNamur), Namur, Belgium; 3 CNRS, UPR3079 CEMHTI, Orléans, France; 4 Université d’Orléans, Polytech Orléans, Orléans, France; 5 ICTEAM Université catholique de Louvain, Louvain-la-Neuve 1348, Belgium

## Abstract

Graphene has emerged as a promising material for infrared (IR) photodetectors and plasmonics. In this context, wafer scale epitaxial graphene on SiC is of great interest in a variety of applications in optics and nanoelectronics. Here we present IR reflectance spectroscopy of graphene grown epitaxially on the C-face of 6H-SiC over a broad optical range, from terahertz (THz) to mid-infrared (MIR). Contrary to the transmittance, reflectance measurements are not hampered by the transmission window of the substrate, and in particular by the SiC *Reststrahlen* band in the MIR. This allows us to present IR reflectance data exhibiting a continuous evolution from the regime of intraband to interband charge carrier transitions. A consistent and simultaneous analysis of the contributions from both transitions to the optical response yields precise information on the carrier dynamics and the number of layers. The properties of the graphene layers derived from IR reflection spectroscopy are corroborated by other techniques (micro-Raman and X-ray photoelectron spectroscopies, transport measurements). Moreover, we also present MIR microscopy mapping, showing that spatially-resolved information can be gathered, giving indications on the sample homogeneity. Our work paves the way for a still scarcely explored field of epitaxial graphene-based THz and MIR optical devices.

Versatile is probably the most appropriate word to qualify the properties of graphene, this one-atom-thick crystal made of carbon^1^,^2^,^3^. For example, changing the number of graphene layers, their stacking, the chemical or electrostatic environment, or the substrate on which graphene is lying has a huge impact on graphene electronic structure: the band gap, carrier type, density and mobility can be tuned at will over extremely large ranges^3^. In turn, this has consequences on electron transport, which has been extensively used since the early days of graphene research to observe and exploit this impressive tunability, e.g. in transistor-like configurations^1^,^2^. The optical properties of graphene are also a sensitive probe of the electronic band structure:^4^,^5^ depending on the considered frequency range, they are determined either by intraband (in the THz and far-IR range), or interband (from MIR to visible range) electronic transitions, as well as by the emergence of a collective electronic excitation (plasmon). New graphene-based optical and optoelectronic components were therefore proposed in the last few years, such as tunable filters, optical modulators or plasmonic metamaterials in the IR regime, taking advantage of the tunability of graphene electronic structure to control its optical properties^3^,^5^,^6^.

As a prerequisite for harnessing the peculiar properties of graphene in such applications, it is mandatory to devise accurate methods to perform IR spectroscopy of graphene on different types of substrates of interest. For epitaxial graphene, i.e. graphene obtained by decomposition of SiC at high temperature^7^, THz to far-IR (FIR) transmission spectroscopy allows to extract carrier density and mobility from a fit to the Drude model^8^,^9^. However, IR transmission measurements are in principle limited to the transparency window of the underlying SiC substrate^7^,^8^,^9^,^10^. This prevents probing graphene properties over a large portion of the MIR range, typically between 85–120 meV (685–968 cm^−1^) and around 200 meV (1613 cm^−1^), due to the phonon-related absorption bands in the SiC substrate^10^,^11^. Moreover, in most applications involving graphene transfered on top of a Si substrate, highly doped Si wafers are preferred, so that graphene carrier density can be tuned using electrostatic gating in a back-gate configuration. This also prevents optical measurements in the transmission configuration due to the substrate opacity. Optical studies in the reflection mode overcome this limitation and can yield a better accuracy, depending on the substrate properties^12^. Moreover, it gives access to the whole spectral range (THz and MIR in particular), but the technique has only been scarcely used and explored on graphene up to now^12^,^13^,^14^,^15^,^16^,^17^,^18^, compared to experiments in the transmission mode^6^,^7^,^8^,^9^,^10^,^11^,^19^. Up to our knowledge, there is no detailed experimental analysis of the optical reflection properties of epitaxial graphene in the THz-MIR ranges. In the present paper, we report an experimental study of the optical reflection of large area epitaxial graphene spanning a broad range of experimental parameters (number of layers, doping level, sample homogeneity). The optical response of the system air/graphene/SiC is analyzed by computing the reflectance spectra using the complex optical conductivity of graphene. Accurate information on the number of layers, doping level (Fermi energy) and mobility (scattering time) can be obtained by considering simultaneously both the THz and MIR ranges in the analysis. We also compare our results to parameters derived from other techniques (Raman spectrocopy, XPS, electron transport). Finally, the IR reflection microspectroscopy is used to extract spatially-resolved information, on the scale of several tens of micrometers, so that it can be used as a reliable non-destructive probe to map the homogeneity of the relevant material properties on wafer-scale graphene.

## Results

### Micro-Raman Spectroscopy

**Figure 1:**
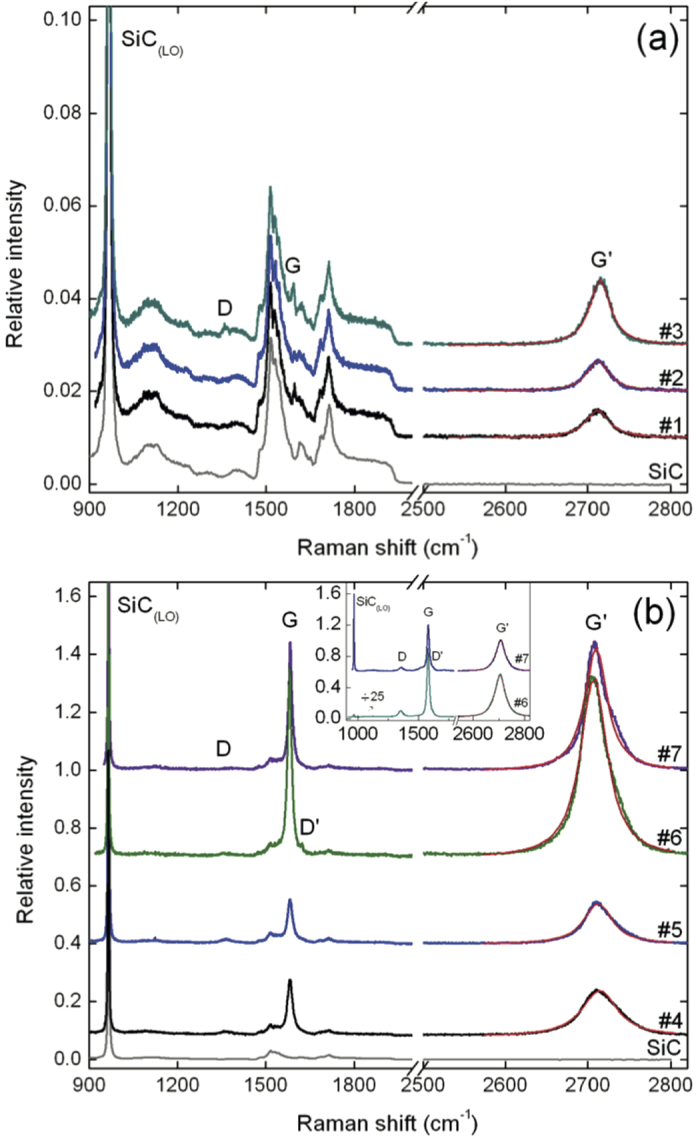
Raman spectra of the FEG (**a**) and MEG (**b**) samples, showing the main Raman features, the *D*, *G* and *G’* bands, taken with a laser wavelength of 514 nm. For the sake of clarity, the spectra of the graphene samples are vertically shifted by 0.01 (Fig. 1a) and 0.3 (Fig. 1b). Inset: Representative Raman spectra of dark inclusions found in samples #6 and #7. In (**a**,**b**), the red lines are Lorentzian fits to the G’ peak.

The micro-Raman spectroscopy results on FEG and MEG samples are summarized in Fig. 1(a,b), respectively. All spectra were normalized with respect to the intensity of the SiC band at 965 cm^−1^, associated with the longitudinal optical (LO) phonon in SiC, and vertically shifted for clarity. The Raman scattering spectra confirm that graphene was successfully grown in all samples, as shown by the presence of the characteristic graphene bands *D*, *G*, and *G’* located around ~1362 cm^−1^, ~1581–1596 cm^−1^ and 2704–2714 cm^−1^, respectively^20^,^21^,^22^,^23^. These bands are superimposed on the second-order Raman features of SiC between 1450–1750 cm^−1^, less pronounced for the thicker MEG samples. In the MEG samples, the *G* and *G’* bands are more intense, indicating a larger number of layers. In all samples (except samples #6 and #7 - see below), the shape of the *G’* band is well fitted by a single Lorentzian curve, as shown in Fig. 1. This indicates that no or few graphitic inclusions are present, and therefore the graphene layers are electronically decoupled due to rotational faults (no AB stacking)^8^,^21^, as expected for our samples^24^,^25^,^26^. For the FEG and MEG samples, the full width at half maximum (FWHM) of the G and G’ bands varies respectively within 26–35 cm^−1^ and 28–52 cm^−1^, in good agreement with previous studies^20^,^21^,^22^,^23^. The spectra of samples #6 and #7 are similar to that presented by Faugeras *et al.* for a thick graphene stack with 70–90 layers^21^. Moreover, a *D’* band appears (~1623 cm^−1^) in the spectrum of sample #6, which indicates a larger density of defects^23^. This can be attributed to a larger disorder, higher amount of grain boundaries^26^, or edges effects. The relative intensity of the *D* band varies among the samples, reflecting different disorder densities in the epitaxial graphene layers. Moreover, within the same sample, the relative intensity of the *D*, *G* and *G’* bands and the FWHM of the G’ band varies (not shown), an indication of local variations in the number of layers and/or doping level and mechanical constraints. Furthermore, some dark inclusions are present in the MEG samples #6 and #7. Two representative spectra measured at the location of these inclusions are shown in the inset of Fig. 1b. The *D* peak is more prominent, together with a *D’* band appearing as a shoulder around 1623 cm^−1^. By comparing the intensity of the graphene features with the LO phonon of the SiC substrate we can infer that these inclusions are a stack of multilayers, thicker in sample #6 than in sample #7. Nevertheless, the *G’* peak is almost single Lorentzian, showing no clear signature of graphitic residues as in earlier studies^21^,^22^.

### Optical response of graphene

**Figure 2:**
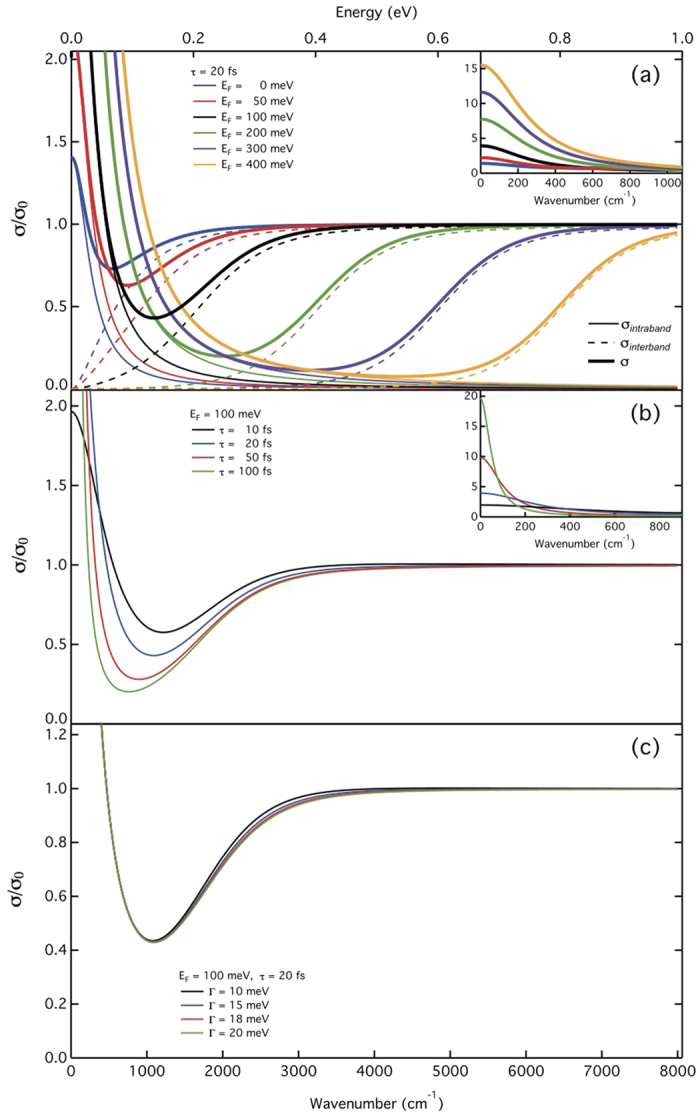
Real part of the optical conductivity of single layer graphene as a function of the Fermi level *E*_*F*_ (**a**), the carrier scattering time τ (**b**), and the broadening of the interband transition Γ (**c**). Insets: optical conductivity in the low wavenumber limit. Thin solid lines: intraband conductivity. Dashed lines: interband conductivity. Bold lines: total optical conductivity. In (**a**,**b**), Γ = 18 meV.

The optical properties of monolayer graphene can be fully described by its complex dynamical in-plane optical conductivity *σ*_*1L*_ = *σ*_*intra*_ + *σ*_*inter*_, which includes both intraband and interband processes^9^,^27^,^28^,^29^ (see Supplementary Note 1). The relative influence of several parameters (Fermi energy *E*_*F*_, carrier scattering time τ and thermal broadening Γ) on graphene optical conductivity in the THz-MIR range can be visualized in Fig. 2. In Fig. 2a, the real part of the total optical conductivity *σ*(*ω*) and of both *σ*_*intra*_(*ω*) and *σ*_*inter*_(*ω*) are shown for varying *E*_*F*_. The computed curves are normalized by *σ*_*0*_ = *e*^2^/4*ħ*. The interband threshold is clearly observed at *ħω* ~ 2*E*_*F*_, and the total conductivity *σ*(*ω*) exhibits significant variations over the full THz and MIR ranges, except above the interband threshold, where *σ*(*ω*) ~ *σ*_*0*_. The inset shows *σ*(*ω*) in the THz range, dominated by the intraband Drude-like absorption. In Fig. 2b,c *σ*(*ω*) was computed for a fixed Fermi level (*E*_*F*_ = 100 meV) while varying τ and Γ, respectively. Increasing τ impacts *σ*(*ω*) in the THz-MIR range, as shown in Fig. 2b. Therefore, a consistent analysis must consider both intra- and interband transitions, over the entire THz-MIR region, in particular in quasineutral graphene. On the other hand, increasing the broadening Γ (Fig. 2c) only smoothens the onset of the interband transition. At room temperature, thermal broadening is usually dominant. Therefore, unless stated otherwise, we have set Γ = 18 meV as obtained by Kuzmenko *et al.*^14^, *i.e.* a value close to the thermal energy *k*_*B*_*T* at room temperature.

Note that in most previous studies on the optical response of epitaxial graphene the intra- and interband contributions were treated separately, mostly due to the difficulty to gather data over the full spectral range, e.g. due to the substrate absorption in particular ranges^8^,^9^,^11^. One can observe that considering both transitions simultaneously, over the full range from THz to MIR leads to a better estimation of all parameters, since both *σ*_*inter*_ and *σ*_*intra*_ can have significant contributions over the full spectral range.

### Reflectance of epitaxial graphene

**Figure 3:**
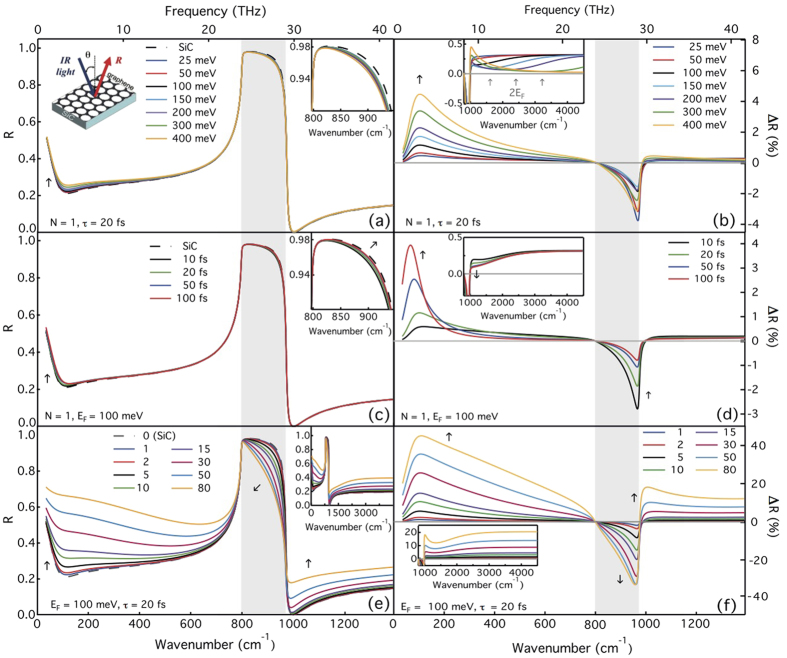
Computed reflectance of FEG/MEG on the SiC substrate for varying the Fermi level *E*_*F*_ (**a**), the carrier scattering time τ (**c**) and the number of graphene layers *N* (**e**). The respective differential reflectance ΔR plots are presented in (**b**,**d**,**f**). Arrows point in the direction of increasing *E*_*F*_, τand *N*. Gray area indicates the *Reststrahlen* band. The upper left inset in (**a**) shows the experimental geometry. The inset in (**b**) shows the interband threshold *2E*_*F*_.

The optical reflectance of graphene layers on a SiC substrate was computed in the framework of the thin film approximation, as shown schematically in the inset of Fig. 3a. We use the appropriate electromagnetic boundary conditions at the interfaces and the graphene layer is described by its complex dynamical optical conductivity *σ*_1L_(*ω*)^30^ (see Methods).

To investigate on the impact of graphene on the overall optical response, we have computed the reflectance of air/SiC and air/graphene/SiC for varying *E*_*F*_, *N*, τ (Fig. 3a-c-e). In each set of spectra, the other parameters are kept constant, and all curves were computed for T = 300 K and Γ = 18 meV. Figure 3b-d-f present the respective differential reflectance spectra, given by Δ*R* *=* *R*_*SiC*_ − *R*, where small variations are more visible than in the raw *R* data^15^. As shown in Fig. 3a-c-e, varying *E*_*F*_, *N* and τ produce significant changes in the optical response in the THz and MIR ranges. For instance, in the case of a graphene monolayer, increasing *E*_*F*_ yields a larger reflectance in the THz region, due to the intraband transition (carriers absorption). In the MIR region, the optical response depends both on intraband and interband transitions, in particular in the *Reststrahlen* band. For a scattering time τ = 20 fs (which yields typical mobilities of 400–1000 cm^2^/Vs in the doping ranges considered here), the reflectance presents a different trend, as detailed in the upper right inset of Fig. 3a when increasing *E*_*F*_ and considering the *Reststrahlen* region, *R* first increases (for *E*_*F*_ < 150 meV) and then decreases (for *E*_*F*_ > 150 meV). This is due to the intraband transition in the low frequency range, and the interband threshold at higher frequencies: below 2*E*_*F*_, the main contribution to the optical conductivity of graphene arises from the carriers absorption. The latter effect is better visualized in Fig. 3b: the absolute value of Δ*R* gradually decreases in the MIR range (in the *Reststrahlen* region), when increasing *E*_*F*_ up to *E*_*F*_ ~ 150 meV and rises again when *E*_*F*_ increases above this value. The inset of Fig. 3b shows the evolution of Δ*R* in the vicinity of the interband threshold (~2*E*_*F*_) when *E*_*F*_ is varied in the range 25–400 meV. The effect of increasing τ is shown in Fig. 3c,d. The main changes occur in the *Reststrahlen* region and in the low frequency limit of the THz range due to the intraband transitions.

**Figure 4:**
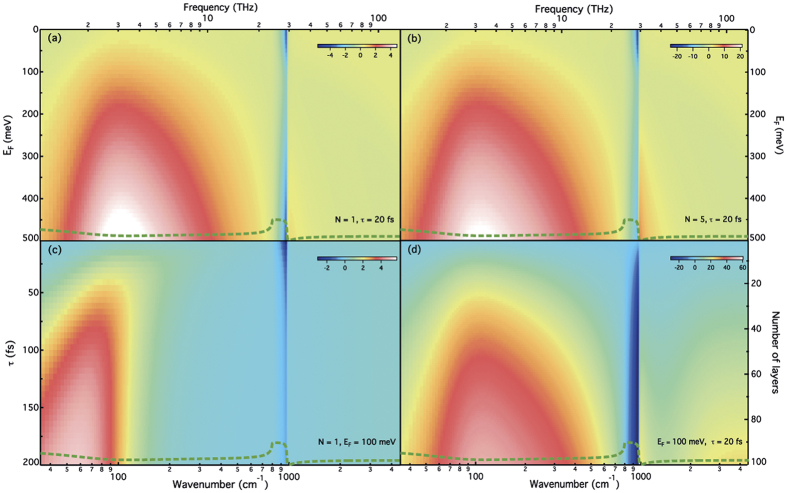
Color plots of the computed differential reflectance (%) of graphene on SiC for varying the Fermi level *E*_*F*_ for monolayer (**a**) and for a stack of 5 multilayers (**b**), the scattering time τ for a monolayer (**c**) and the number of layers *N* (**d**). The reflectance of the SiC substrate is shown as a guide to the eyes.

Figure 3e presents the obtained *R* for increasing *N*. From monolayer (*N* = 1) to FEG/MEG, *R* increases in the THz and MIR regions, with an opposite trend in the *Reststrahlen* band. The optical contrast increases with increasing *N*, with respect to the SiC substrate, leading to remarkable changes within this region. The latter feature alone can be used to assess the formation of graphene and evaluate the number of graphene layers. As reported recently^17^ and in agreement with our calculations, a small contrast is obtained in the case of monolayer epitaxial graphene. As shown in a previous study^31^, the differential reflectance in the MIR can also be used to estimate *N*, analogous to transmission measurements. For highly doped graphene, this should be performed in the NIR region due to the interband threshold (see inset of Fig. 3b). A direct visual assessment of the sensitivity of the IR reflection technique to the different parameters of the graphene layers is provided by the color plots shown in Fig. 4a–d. The reflectance of SiC is displayed on each color plot as a guide to the eyes. Thanks to such plots, one can directly translate the Δ*R* measurement sensitivity into the sensitivity on the measurement of *E*_*F*_, τ and *N*. In particular, one can stress again that the analysis of the full THz/MIR range will improve accuracy in determining graphene properties as each parameter affects distinct regions of the optical reflectance, in a qualitatively different way. Comparing the monolayer response with that of a FEG with a 5 layers stack (Fig. 4a,b, respectively), we can infer that the features in the THz-MIR range remain unchanged (for constant *E*_*F*_ and τ), although the contrast increases with the number of layers. Therefore slight variations in *E*_*F*_, *N* and τ will result in a unique optical spectrum that can be distinguished from those obtained using different sets of parameters, as will be demonstrated below.

**Figure 5:**
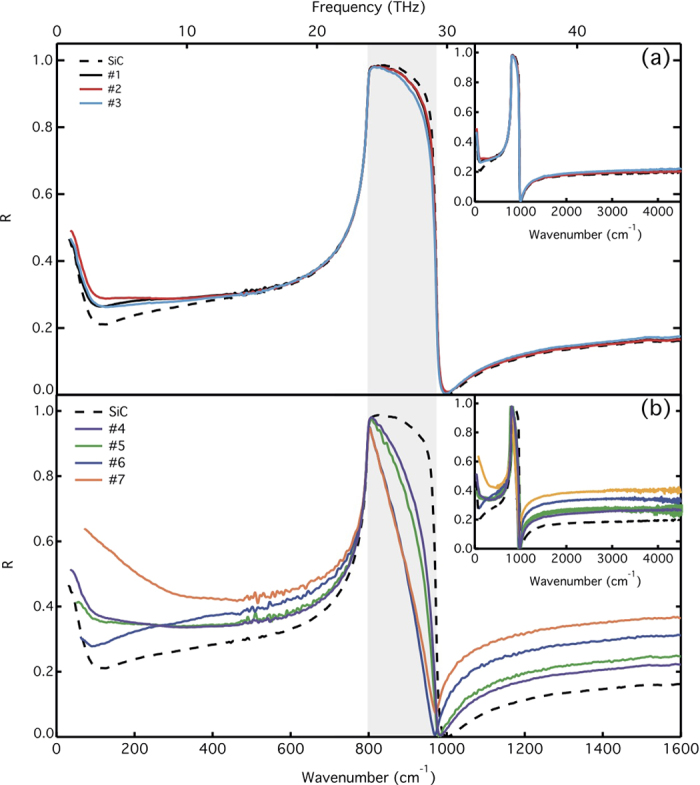
THz and MIR reflectance of the SiC substrate and epitaxial graphene. (**a**) FEG samples. (**b**) MEG samples. Inset: Reflectance curves in the entire measured range.

**Figure 6:**
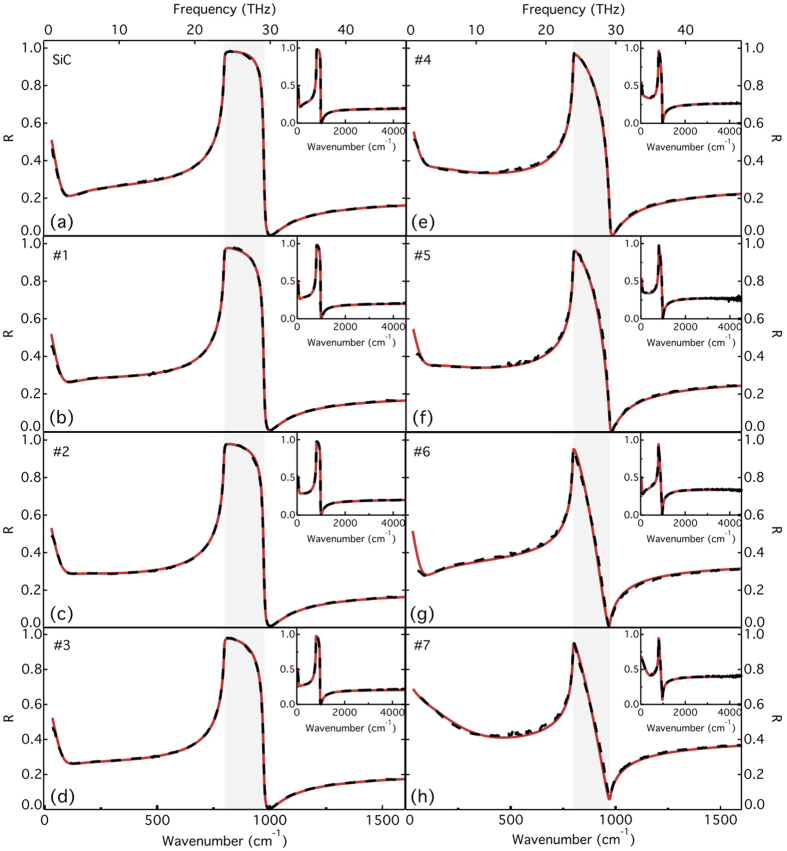
THz and MIR reflectance of the SiC substrate (**a**), FEG (**b–d**) and MEG (**e–h**) samples. Dashed lines: experimental data. Solid lines: model. The inset shows the same curves from the THz to the MIR range.

To compare with the theoretical predictions, we present in Fig. 5a,b the THz-MIR reflectance spectra of SiC (dashed line) and of FEG/MEG samples with different number of layers. The SiC spectrum is in good agreement with previous reports^17^,^32^,^33^,^34^. As expected from the model, *R* changes markedly with varying *N*. Moreover, the shape of the curves in the THz range is qualitatively different for each sample, an indication that the carriers properties are distinct. The analysis of the experimental data was accomplished by fitting the reflectance model to the given spectra over the full THz-MIR ranges. We present the obtained fitting curves in Fig. 6a–h, along with the experimental data. One first notes that the model can accurately reproduce the optical response of the air/graphene/SiC system over the whole investigated range, in a large variety of samples. Small differences between data and computed spectra are attributed to samples inhomogeneity, i.e. local variations in the carriers density, mobility and/or average number of layers. We will show below that the XPS and IR microscopy results confirm the existence of spatial variations. The fitting curves in Fig. 6a–h correspond to the best-fit parameters summarized in Table 1, along with the number of layers estimated from XPS measurements, which closely matches the values obtained from the fit to the IR reflectance spectra. The presented XPS values correspond to measurements performed on two distinct regions in the samples. In both IR and XPS measurements, we obtain an average thickness, which can vary within the same sample due to the growth kinetics. This can explain small differences in the results obtained with the two techniques. Note that XPS can not provide accurate estimates for the number of layers when the thickness of the MEG film is above ~10 nm, and therefore we could not determine *N* from XPS for samples #6 and #7. Nevertheless, it corroborates with the fits to the IR reflectance data: we indeed find *N* > 35.

### IR microscopy

**Figure 7:**
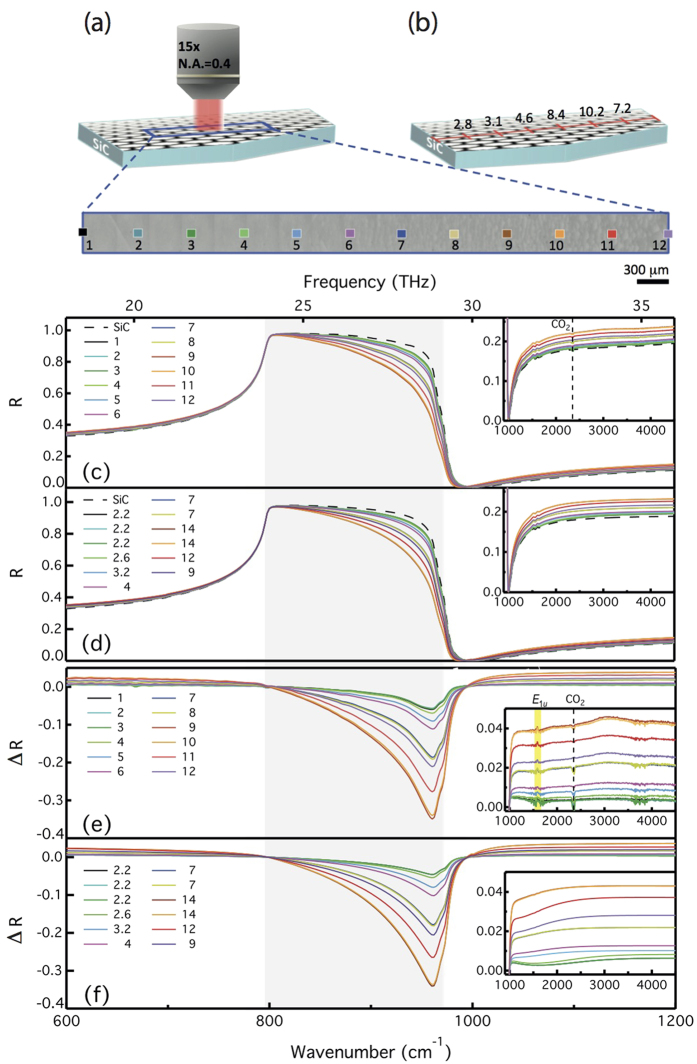
IR microscopy of sample #3. (**a**) Optical image, showing the changing microstructure along the sample. (**b**) Number of graphene layers measured by XPS as a function of the position along the sample length (red line). (**c**) Experimental IR reflectance microscopy on each spot at locations indicated in (**a**). (**d**) Fit to the experimental spectra in (**c**). The extracted number of layers is indicated on the graph. Experimental (**e**) and computed (**f**) differential reflectance spectra at locations indicated in (**a**). Inset: Reflectance (**c,d**) and differential reflectance (**e,f**) spectra above 1000 cm^−1^. The shaded area in the inset of (**e**) indicates the graphene phonon *E*_*1u*_.

As shown above, the remarkable changes in the *Reststrahlen* band are closely related to the properties and number of graphene layers. This can be demonstrated by examining local IR reflectance data from a FEG sample with a heterogeneous number of layers *N*. Due to its trapezoidal shape, sample #3 was subject to an intentional temperature gradient during graphene growth. We could therefore anticipate that this would give rise to a gradient in *N* along the sample length. Figure 7a shows schematically the shape of sample #3 and an optical micrograph of the area under investigation. One can readily observe changes in the optical contrast from left to right (i.e. from the low- to high-temperature region), corresponding to changes in *N*. We now show that this gradient can be measured thanks to a mapping of reflectance spectra in the MIR range, a rapid and non-invasive method. The number of graphene layers is usually estimated from data in wavenumber range above 3200 cm^−1^ 12. We have shown previously that the number of layers also strongly impacts the reflectance in the MIR region and that the IR response, in particular in the *Reststrahlen* band, can be used to estimate *N*.

IR spectra taken at the positions indicated by the numbered colored squares on Fig. 7a are displayed in Fig. 7c (with the corresponding color). There is a gradual spatial evolution in the spectra, particularly visible in the *Reststrahlen* band: the reflectance is decreasing when going from the left- to the right-hand side of the sample. Fitting the data to the reflectance model yields the spectra presented in Fig. 7d: *N* is gradually varying from 2.2 to 14 layers between the two ends of the measured region (see Methods and Supplementary Note 3). This result corroborates with the thickness derived from the XPS mapping (spot size of 1 × 1 mm^2^) performed on the same sample, as shown in Fig. 7b. Here we used an IR aperture of hundreds of square micrometers (170 × 170 μm^2^) to approach the experimental conditions of the XPS measurements (1 mm^2^ spot). Note that for the spectral region analyzed here and our experimental setup configuration, the spatial resolution could be improved to 25–30 μm.

Besides being a rapid and non-invasive tool to differentiate between epitaxially grown FEG or MEG samples, it also offers perspectives for mapping the homogeneity of the electronic properties across graphene samples. *E*_*F*_ can be estimated from the threshold in the MIR region. The same kind of analysis can be performed on CVD graphene transferred to other opaque or transparent substrates e.g. Si/SiO_2_ or quartz. Note that, in our case, the detector used in the IR mapping has a higher sensitivity than the one used in the classical IR spectroscopy. We can thus observe that when *N* increases, two features appear: a peak at 1584 cm^−1^, and a broad band centered around 3200 cm^−1^ (inset of Fig. 7d) The first peak is related to the graphene in-plane optical TO phonon *E*_1*u*_ (not IR active in monolayer graphene)^14^. The second one is an indication of a weak electronic coupling between graphene layers in bilayers inclusions with AB (Bernal) stacking due to rotational faults, as shown recently in MEG samples^35^. This absorption occurs at an energy corresponding to the interlayer coupling parameter *γ*_1_ ~ 0.37 eV (~2984 cm^−1^) in bilayer graphene^8^,^15^,^31^,^36^. Nevertheless, it represents a weak absorption, and it is only 5% higher than the graphene universal absorption for the thicker layers (e.g., spots #9–12). The coupling is lower than the one observed in other epitaxial samples^8^. This finding shows that IR microscopy is very sensitive to the interlayer coupling in epitaxial graphene and it has the advantage of probing the whole stack of graphene layers, and not only the top few layers.

## Discussion

For the sake of comparing the results obtained from the IR analysis, an independent measurement of *E*_*F*_ and τ was obtained through magnetotransport experiments on the FEG sample #1. The Hall coefficient was measured in a Hall bar geometry at low temperature (4.2 K) to avoid parallel conduction through the substrate^37^,^38^. From this result, we estimate *E*_*F*_ = 172 meV (with *n*-type charge carriers), i.e. in excellent agreement with *E*_*F*_ = 170 ± 5 meV found from the analysis of IR spectra. In addition, measurement of the sheet resistance at zero magnetic field yielded τ = 12.5 ± 3 fs using the Einstein relation^39^, i.e. in good agreement with the value obtained through the IR reflectance measurements (τ_*IR*_ = 16 ± 2 fs), considering the differences in experimental conditions (the electron transport experiment probed a few micrometers-squared area).

**Figure 8:**
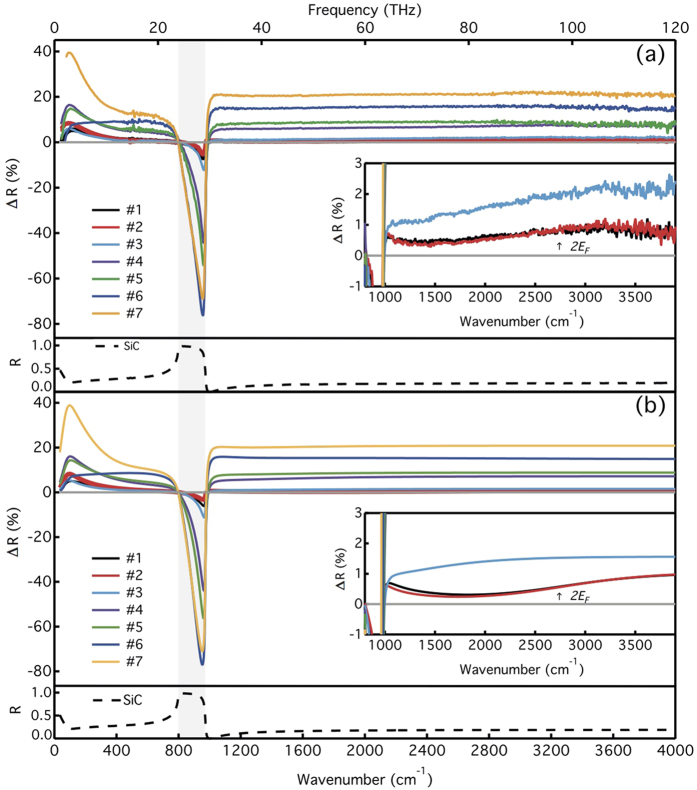
Differential reflectance spectra of the studied samples, together with SiC reflectance. (**a**) Experimental data. (**b**) Model. Inset: The interband threshold at 2*E*_*F*_ in the MIR range for samples #1 and #2.

From the computed Δ*R* (Figs 3d and 4c), we could already deduce that the reflectance in the THz region should be very sensitive to the charge carriers mobility. This is true even when the number of layers gets larger, as experimentally illustrated in Fig. 8a, showing the experimental differential reflectance Δ*R.* As shown in Fig. 8b, there is a good agreement between the experimental and calculated spectra. Comparing the spectrum obtained for sample #6 (low mobility) with those of the other samples, one can note that the latter exhibit a rather flat dependence of Δ*R* in the THz region, indicative of a poor carrier mobility (which is also consistent with the presence of *D* and *D’* bands in the Raman spectrum in Fig. 1), while the other samples exhibits an increasing Δ*R* in that spectral region, a signature of a higher mobility.

## Conclusions

In summary, IR reflectance proves to be a quantitative, versatile and non-invasive tool to investigate the electronic properties of graphene. The number of epitaxial graphene layers has a strong influence on the optical response of graphene/SiC samples over the full THz-MIR range, and therefore a rapid investigation can already provide an evaluation of the number of layers even for a large number of layers. The IR reflectance was modelled over the entire THz-MIR range, based on the substrate dielectric function and graphene optical conductivity. Fitting IR reflectance data using this model, we obtained a good estimate of the quality and electronic properties of the grown graphene layers, corroborating results obtained using other techniques. Moreover, IR microscopy was shown to be a fast and reliable local probe technique to map the variations of the carriers properties and the number of graphene layers, as well as interlayer interactions. IR reflectance spectroscopy could be advantageous for exploring graphene properties and optical devices on a variety of substrates in the THz to the near-infrared range.

## Methods

### Sample preparation and characterization techniques

In this work, we examine graphene layers grown epitaxially by thermal decomposition on the C-terminated face of a hexagonal silicon carbide substrate - 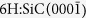, in ultrahigh vacuum (UHV) via resistive heating as described previously^24^,^25^. Varying the synthesis parameters (growth time and temperature), few-layer to multilayer epitaxial graphene can be obtained (FEG and MEG, respectively). In order to study the effect of graphene properties on the THz-MIR optical response, a set of seven FEG to MEG samples were grown using intentionally different conditions.

IR spectroscopy was performed from FIR or THz up to the MIR range in a Fourier-transform infrared spectrometer (FTIR) Bruker IFS 113v under vacuum (~1 mBar) equipped with a liquid helium-cooled Si bolometer, mylar (6 μm, 12 μm) beamsplitters, a deuterated-triglycine sulfate (DTGS) detector, a KBr beamsplitter, and a silicon carbide light source (globar). The measurements covered the spectral range between 35 and 4500 cm^−1^, corresponding approximately to 1–135 THz (or 285–2.2 μm), where 1 THz = 33.3 cm^−1^ = 300 μm = 4.1 meV. The IR beam aperture diameter on the sample was set between ~1.25 and ~2.5 mm^2^, depending on sample dimensions, and optimized to obtain the best signal-to-noise ratio. All measurements were performed at near-normal incidence (θ ~ 8°). A bare Al mirror was used as reference. In addition, IR spectroscopy mapping was employed to investigate sample homogeneity. In this case, the measurements were performed under ambient conditions using an IR microscope (Bruker Hyperion 3000) coupled to a FTIR spectrometer (Bruker Vertex 80v) under vacuum (~1 mBar). A liquid nitrogen-cooled mercury cadmium telluride (MCT) detector, a KBr beamsplliter and a globar source were used, covering the MIR and NIR regions, between 400 and 7000 cm^−1^ (12–210 THz or 25–1.4 μm). The IR beam was focused on the sample by a Cassegrain 15x IR objective (NA = 0.4) with a square beam-aperture of 170 × 170 μm^2^. An Au-coated mirror was used as reference. In all measurements the spectral resolution was set to 4 cm^−1^. The systematic error in the relative value of the reflectance (for R = 0.985) is around 1% and 2–3% for the IR spectroscopy and microspectroscopy, respectively. Micro-Raman measurements were performed in a LabRam HR spectrometer (Jobin Yvon - Horiba), in a confocal back-scattering setup with a 514 nm (2.41 eV) laser beam focused by a 100× optical objective (NA = 0.95). A high-resolution diffraction grating of 1800 grooves/mm was used. The laser power was kept below 1 mW to avoid damaging of the graphene layers.

Angle-resolved X-ray photoelectron spectroscopy (XPS) was performed using an Escalab 250Xi instrument from Thermo Scientific and the Al K_α_ line. The number of graphene layers was estimated by the integrated intensity ratios of the C1s core level for graphene (~284.5 eV) and SiC (~282.7 eV), as described in Biederman *et al.*^40^ (see Supplementary Note 2). The presented results are the average for four different photoelectron emission angles from the surface normal (0°, 17.5°, 35° and 52.5°).

### Optical reflectance model

In the case of two dielectric media 1 (vacuum) and 2 (SiC substrate), as shown schematically in the inset of Fig. 3a in the main text, in the near-normal incidence configuration, the total specular reflectance *R* between the two media separated by the graphene layer is given by:^30^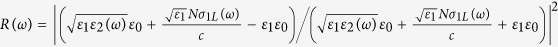, where *σ*_*1L*_ is the conductivity of monolayer graphene, *c* is the speed of light in vacuum, *ε*_*0*_, *ε*_*1*_ and *ε*_*2*_ are, respectively, the permitivity of vacuum, and the relative dielectric functions of vacuum (*ε*_*1*_ = 1) and of the SiC substrate. We have included the number of graphene layers *N* in the model by considering that rotational disorder between layers is large enough so there is no Bernal (AB) stacking^8^,^11^,^35^,^41^,^42^,^43^. Scanning tunnelling microscopy (STM) analysis of samples produced with the same method as the one used here indeed presented Moiré patterns due to the misalignment between adjacent layers^25^. For epitaxial graphene grown on the C-face of SiC it is indeed known that AB stacking or bilayer inclusions are only present in small portions of the sample as rotational faults^8^,^11^,^35^,^41^,^42^. Nevertheless, the graphene multilayers present electronic features typical of isolated graphene monolayer^41^,^43^. As a consequence, one can assume that individual layers are electronically decoupled, and that each layer exhibits the properties of a graphene monolayer. In this framework, the optical conductance of FEG/MEG is simply *σ*_*FEG/MEG*_ = *Nσ*_*1L.*_The optical response of SiC is described by a Drude-Lorentz model:^44^,^45^,^46^ 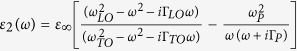, where *ε*_*∞*_ is the high-frequency dielectric constant. The first term in the above equation is the well-known four parameters damped oscillator model, where *ω*_*LO*_(*ω*_*TO*_) and Γ_*LO*_(Γ_*TO*_) denotes respectively the frequency and damping rate of longitudinal (LO) and transverse (TO) optical phonons. The second term describes the Drude absorption due to the free carriers in the n-doped 6H:SiC. All the main features of the reflectance spectrum of SiC were well reproduced using the following fixed parameters: *ε*_*∞*_ = 6.55, *ω*_*TO*_ (*ω*_*LO*_) = 797.7 (970.7) cm^−1^, Γ_TO_(Γ_LO_) = 2.3 (5.6) cm^−1^, *ω*_*P*_ = 103 cm^−1^ and Γ_*P*_ = 90  cm^−1^, in good agreement with previous reports^32^,^33^,^34^,^45^,^47^,^48^. For the IR microscopy data, a semi-quantum dielectric function^49^,^50^ was used to fit the SiC response (see Supplementary Note 3). The FEG/MEG average carrier density *n* and average mobility μ are respectively given by *n* = (N/π)(*E*_*F*_/*ħv*_F_)^2^ and *μ* = *ev*_F_τ/*ħ*(π*n*)^1/2^ 8,13,51, where v_F_ is the Fermi velocity in graphene (~10^6^ m/s). For epitaxial graphene, it was demonstrated that only the first few graphene layers, closer to the substrate, and the outermost layer in MEG are doped, whereas the remaining ones are quasi-neutrals^52^. Here, we assume a constant Fermi energy among all the layers. Therefore for the MEG samples, the extracted carrier concentration should accordingly be redistributed on the 3–4 layers that are closer to the substrate.

## Additional Information

**How to cite this article**: Santos, C. N. *et al.* Terahertz and mid-infrared reflectance of epitaxial graphene. *Sci. Rep.* **6**, 24301; doi: 10.1038/srep24301 (2016).

## Supplementary Material

Supplementary Information

## Figures and Tables

**Table 1:** Best-fit parameters obtained from the IR reflectance model for each FEG/MEG sample.

Sample #	*N*_*IR*_	*N*_*XPS*_ (±20%)	*E*_*F*_ (meV)	τ (fs)	N(10^12^ cm^−2^)	μ (cm^2^/Vs)
1	3.3 ± 1.0	2.4 2.0	170	16	7.0	518
2	3.4 ± 1.0	1.7 1.9	170	27	7.2	862
3	5.0 ± 1.0	3.0 3.5	65	29	1.6	1996
4	24 ± 3	19.1 22.4	55	23	5.3	854
5	30 ± 3	29.3 21.7	38	20	3.2	961
6	53 ± 4	>35	40	5	6.2	172
7	82 ± 6	>35	25	38	3.8	1680

The two results given for *N*_*XPS*_ correspond to two different regions of the samples.
