# Supplementary material for: Terahertz and mid-infrared reflectance of epitaxial graphene
Source: Sci Rep. 2016 Apr 22;6:24301. doi: 10.1038/srep24301 (PMC4840310; doi:10.1038/srep24301)
Supplement: Supplementary Information [file srep24301-s1.pdf]

SUPPLEMENTARY INFORMATION for

"Terahertz and mid-infrared reflectance of epitaxial graphene"

Cristiane N. Santos,[†,*] Frédéric Joucken,[⊥] Domingos De Sousa Meneses,[‡,§] Patrick Echegut,[‡] Jessica Campos-Delgado,[#] Pierre Louette,[⊥] Jean-Pierre Raskin,[#] Benoit Hackens[†]

[†]IMCN/NAPS

Université catholique de Louvain

Louvain-la-Neuve 1348, Belgium

[⊥]Research Center in Physics of Matter and Radiation (PMR), University of Namur (FUNDP), Namur, Belgium

[‡]CNRS, UPR3079 CEMHTI, Orléans, France

[§]Université d'Orléans, Polytech Orléans, Orléans, France

[#]ICTEAM

Université catholique de Louvain

Louvain-la-Neuve, 1348 Belgium

*Address correspondence to nsantos.cristiane@gmail.com; cristiane.nascimento@uclouvain.be

Supplementary Note 1. Optical conductivity of graphene

The general expression of the optical conductivity of graphene can be derived from the Kubo formula or within the random-phase approximation (RPA):[1-4]

$\sigma(\omega) = \sigma_{intraband} + \sigma_{intreband}$ with

$$\sigma_{\mathrm{int}raband}(\omega) = i\frac{e^2/\pi\hbar^2}{\omega + i/\tau}\int_0^\infty dE \times \left[f(E-E_F) + f(E+E_F)\right] \quad \text{(S1)}$$

$$\sigma_{\mathrm{int}erband}(\omega) = i\frac{e^2\omega}{\pi}\int_0^\infty \frac{dE}{(2E)^2 - (\hbar\omega + i\Gamma)^2} \times \left[f(E-E_F) - f(-E-E_F)\right] \quad \text{(S2)}$$

where both intra- and interband transitions are taken into account. Here e is the electron charge, $\hbar$ the reduced Planck's constant and ω the angular frequency. In both equations (S1) and (S2), $f(\pm E \pm E_F) = 1/[1+ exp((\pm E \pm E_F)/k_BT)]$ is the Fermi-Dirac distribution, where E_F is the Fermi energy. The intraband electron-photon processes, due to free carriers absorption, is described by a Drude-like response with a finite carrier scattering time τ. The interband contribution plays the leading role above E_F, due to Pauli blocking, with an absorption threshold at $\hbar\omega \sim 2E_F$ and a broadening given by Γ. Above $2E_F$, *i.e.* typically in the NIR to visible spectral range, the well known intrinsic graphene universal optical conductivity $\sigma_0 = e^2/4\hbar$ dominates, leading to an optical absorption of $\pi\alpha$ = 2.3% that only depends on the fine-structure constant α.[5,6] Analytic expressions can be obtained for limiting cases such as $E_F = 0$ or $T = 0$ K.[6,7] In our case, we have numerically computed the integrals at $T = 300$ K.

Supplementary Note 2. Thickness estimation of graphene layers by X-ray photoelectron spectroscopy (XPS)

The evaluation of the number of layers in the epitaxial graphene samples was performed by high-resolution XPS measurements of the C1s core-level, described as follows.

The FEG/MEG samples consist in several graphene layers on top of a SiC crystal, as schematized in Figure S1 (the distances are taken from ref. 8)

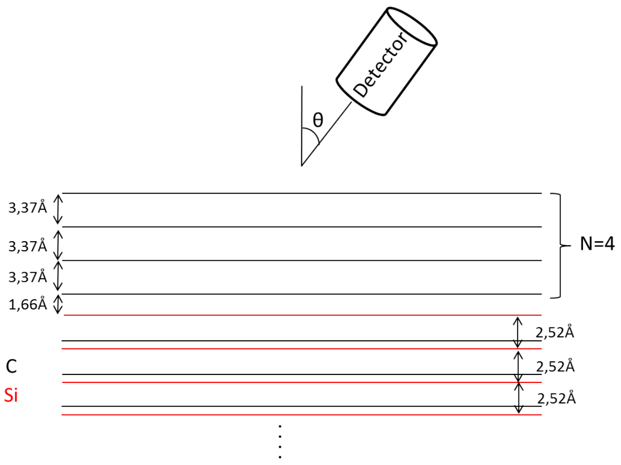

Figure S1: Schematics of the XPS measurement on a FEG (N=4) film on SiC.

Typical high resolution photoelectron spectra of the C1s core-level are shown in Figure S2a for one FEG sample (N ~ 2), and one MEG sample (N ~ 20) in Figure S2b, obtained at different take-off angles (0°, 17.5°, 35°, 52.5° and 70°).

The integrated area of the C1s peak of the graphitic carbon I_{C1s}^{G} is given by

$$I_{C1s}^{G} = \sigma_{C1s}\rho_G\left(1 + e^{\frac{-3.37}{\lambda_{C1s}^{G}\cos\theta}} + \cdots + e^{\frac{-(N-1)3.37}{\lambda_{C1s}^{G}\cos\theta}}\right) = \sigma_{C1s}\rho_G\frac{1-e^{\frac{-N\,3.37}{\lambda_{C1s}^{G}\cos\theta}}}{1-e^{\frac{-3.37}{\lambda_{C1s}^{G}\cos\theta}}} \qquad \text{(S3)}$$

where each term in the sum corresponds to one given graphene layer (attenuated according to its position in the stack of N layers). [9] In the above equation, θ is the take-off angle (cf. Figure S1), ρ_G is the surface density of carbon atoms in a graphene layer, λ_{C1s}^{G} is the inelastic mean free path (IMFP) of the photoelectron ejected from the *C1s* state and travelling in the graphitic film. σ_{C1s} is a proportionality constant, typical of the *C1s* state, which takes into account other parameters influencing the peak intensity

(such as the pass energy of the analyser, the intensity of the X-ray source, the kinetic energy of the photoelectrons, the temperature ...).

Similarly, the intensities for the C1s photoelectrons emitted from the carbon atoms of the SiC substrate can be evaluated as

$$I_{C1s}^{SiC} = \sigma_{C1s}\rho_{SiC}\frac{e^{\frac{-[(N-1)3.37+1.66]}{\lambda_{C1s}^{G}\cos\theta}}}{1-e^{\frac{-2.52}{\lambda_{C1s}^{SiC}\cos\theta}}} \quad \text{(S4)}$$

where ρ_{SiC} is the surface density of carbon atoms in a carbon layer in SiC and λ_{C1s}^{SiC} is the IMFP of the photoelectron ejected from the *C1s* state and travelling in SiC. In equation 2, we assume that ρ_G/ρ_{SiC} =3.15[10] and the IMFP were determined using NIST-SRD71.[11]

The thickness of the epitaxial graphene samples was estimated from the intensity of the C1s photoemission lines. The relative intensitie $I_{C1s}^{G}/I_{C1s}^{SiC}$ was determined from the integrated peak areas, and the number of layers was obtained by comparing this value with the ratio calculated using equations S3 and S4, for each take-off angle. (Note that to gain more confidence in our results, we also determined the number of layers by comparing the peak intensities of the *Si2p* and the graphitic *C1s* states, which lead to similar results.)

As in previous works, [9, 10] there are some sources of uncertainties. We can cite the IMFP and the peak area determination (typical fits are shown on Figure S2 as blue and red dashed curves), as well as the presence of defects (such as grain boundaries) in the samples. One has also to keep in mind that in these samples, the graphene thickness is non-uniform, as shown by STM[12], and PEEM and LEEM measurements.[13,14]

It is known that estimating epitaxial graphene thickness using XPS may lead to accuracies in the order of 1 – 2 layers for few layer graphene samples [8]. Taking into account these elements (IMFP, peak area determination, sample coverage), we reasonably estimate the uncertainty when determining the number of graphene layers

with this method to be roughly around 20%. Note that the two results given in table 1 of the main text for N_{XPS} in some samples correspond to two different regions of the samples.

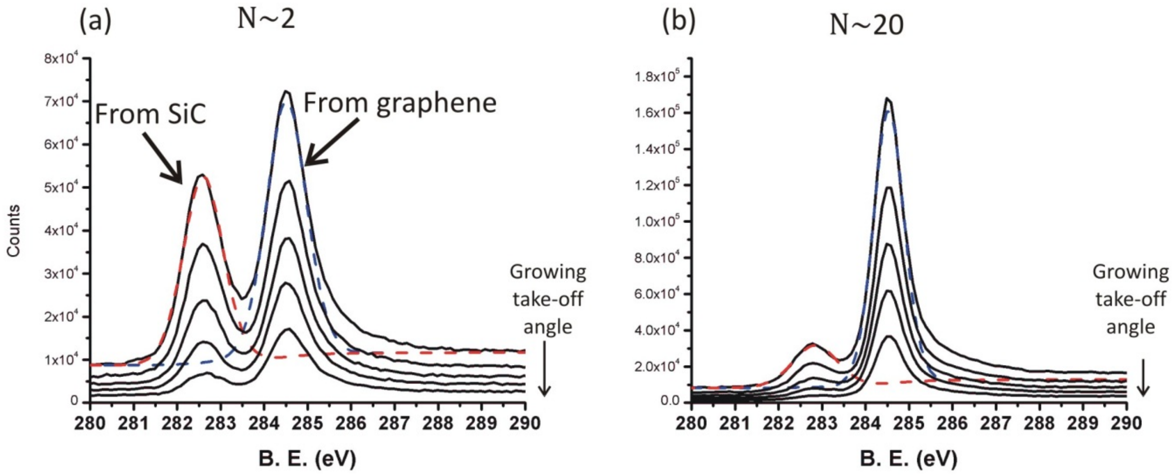

Figure S2: Illustrative *C1s* XPS spectra acquired for different take-off angles (0°, 17.5°, 35° 52.5° and 70°) on a sample with few (~2, a) and many (~20, b) graphene layers. The fits used for the peak area determination are shown on the spectra at $\theta = 0°$ in red and blue for the carbide and graphitic components, respectively.

Supplementary Note 3. Reflectance model for the IR microscopy data

The SiC substrate is an anisotropic material. In consequence, due to its uniaxial crystal anisotropy, the 6H-SiC reflectivity will depend on the angle of incidence and the frequency of the optical phonons depend on the propagation direction in the crystal (*s*- or *p*-polarized light). Therefore, the pure transverse (TO) or longitudinal (LO) character and symmetry of the optical phonons disappears due to mixing effects for incidence angles other than θ = 0° and θ = 90°.[15] In the microscopy measurements, due to the Cassegrain objective, which illuminates

the sample within a solid angle of up to 25° off-normal,[16, 17] a contribution from the $LO_{||}$ phonon mode (*p*-polarization) can be seen around $\omega_{LO||}$ = 964 cm^{-1}.[15,18] We have taken this into account in the model by using a semi-quantum approach [19] to compute the SiC dielectric function, which in turn allows us to better describe the substrate reflectivity including the multiphonon features between 1500-1600 cm^{-1}. This was performed using the freeware FOCUS.[20] The IR microscopy measurements were performed in ambient conditions. Narrow absorption bands appear due to water (1450-1730 cm^{-1} and 3600-3900 cm^{-1}) and CO_2 (2280-2390 cm^{-1}) in the air.

Supplementary Note 4. Raw reflectance data vs. differential reflectance

One should note that in a previous work,[21] a peak centered around 1000 cm^{-1} was identified in a plot of the ratio $R_{Graphene+SiC}/R_{SiC}$. Due to the low substrate reflectivity (R_{SiC} ~ 0 and ε_{SiC} ~1) at this wavelength, computing this ratio can induce systematic errors. In that work, the authors attributed this peak to the formation of a surface plasmon-phonon polariton (SPPhP) mode due to the excitation of graphene plasmons. Nevertheless, the SiC can only support a surface phonon polariton (SPhP) mode in the *reststrahlen* region (797-970 cm^{-1} or ~ 12.5-10.3 μm), where the real part of ε_{SiC} is negative [16,17,22] and not between 8-10 μm as stated by the authors.

References

1. Dawlaty, J. M. *et al*. Measurement of the optical absorption spectra of epitaxial graphene from terahertz to visible. *Appl. Phys. Lett.* **93,** 131905 (2008).

2. Gusynin, V. P., Sharapov, S. G. & Carbotte, J. P. Magneto-optical conductivity in graphene. *J. Phys.: Condens. Matter* **19,** 026222 (2009).

3. Falkovsky, L. A. & Varlamov, A. A. Space-time dispersion of graphene conductivity. *Eur. Phys. J. B* **56,** 281-284 (2007).

4. Falkovsky, L. A. & Pershoguba, S. S. Optical far-infrared properties of a graphene monolayer and multilayer. *Phys. Rev. B* **76,** 153410 (2007).

5. Nair, R. R. *et al. Science* **320,** 5881 (2008).

6. Li, Z. Q. *et al*. Dirac charge dynamics in graphene by infrared spectroscopy. *Nat. Phys.* **4,** 532-535 (2008).

7. Koppens, F. H., Chang, D. E. & de Abajo, F. J. G. Graphene plasmonics: a platform for strong light matter interactions. *Nano Lett.* **11,** 3370–3377 (2011).

8. Hass, J. et al. Structural properties of the multilayer graphene/4H−SiC(000-1) system as determined by surface x-ray diffraction, *Phys. Rev. B* **75**, 214109 (2007).

9. Biedermann, L. B., Bolen, M. L., Capano, M. A., Zemlyanov, D. & Reifenberger, R. G. Insights into Few-layer Epitaxial Graphene Growth on 4H-SiC(000-1) Substrates from STM Studies. *Phys. Rev. B* **79**, 125411 (2009).

10. Hass, J, de Heer, W. A. & Conrad, E. H. The growth and morphology of epitaxial multilayer graphene, *J. Phys.: Condens. Matter* **20**, 323202 (2008).

11. http://www.nist.gov/srd/nist71.cfm

12. Hiebel, F. and Mallet, P. and Magaud, L. & Veuillen, J.-Y. Atomic and electronic structure of monolayer graphene on 6H-SiC(000-1)(3×3): A scanning tunneling microscopy study, *Phys. Rev. B* **80**, 235429 (2009).

13. Johansson, L. I. and Watcharinyanon, S. and Zakharov, A. A. and Iakimov, T. and Yakimova, R. & Virojanadara, C. Stacking of adjacent graphene layers grown on C-face SiC, *Phys. Rev. B* **84**, 125405 (2011).

14. Luxmi and Srivastava, N. and He, Guowei and Feenstra, R. M. & Fisher, P. J. Comparison of graphene formation on C-face and Si-face SiC (0001) surfaces, *Phys. Rev. B* **82**, 235406 (2010).

15. Engelbrecht, F. & Helbig, R. Effect of crystal anisotropy on the infrared reflectivity of 6H-SiC. *Phys. Rev. B* **48**, 15698–15707 (1993).

16. Caldwell, J. D. *et al*. Low-loss, extreme sub-diffraction photon confinement via silicon carbide localized surface phonon polariton resonators. *Nano Lett.* **13**, 3690-3697 (2013).

17. Chen, Y. *et al*. Spectral tuning of localized surface phonon polariton resonators for low-loss mid-IR applications. *ACS Photonics* **1,** 718–724 (2014).

18. Tiwald, T. E. *et al*. Carrier concentration and lattice absorption in bulk and epitaxial silicon carbide determined using infrared ellipsometry. *Phys. Rev. B* **60**, 11464-11474 (1999).

19. De Sousa Meneses, D., Brun, J., Echegut, P. & Simon, P. Contribution of semi-quantum dielectric function models to the analysis of infrared spectra. *Appl. Spectrosc.* **58**, 969-974 (2004).

20. Focus Web Site: http://crmht.cnrs-orleans.fr/pot/software/focus.html.

21. Daas, B. K., Daniels, K. M., Sudarshan, T. S. & Chandrashekhar, M. V. S. Polariton enhanced infrared reflection of epitaxial graphene. *J. Appl. Phys.* **110,** 113114 (2011).

22. Huber, A., Ocelic, N., Taubner, T. & Hillenbrand, R. Nanoscale resolved infrared probing of crystal structure and of plasmon-phonon coupling. *Nano Lett.* **6**, 774–778 (2006).
